# Plasmid composition in *Aeromonas salmonicida subsp. salmonicida* 01-B526 unravels unsuspected type three secretion system loss patterns

**DOI:** 10.1186/s12864-017-3921-1

**Published:** 2017-07-12

**Authors:** Katherine H. Tanaka, Antony T. Vincent, Jean-Guillaume Emond-Rheault, Marcin Adamczuk, Michel Frenette, Steve J. Charette

**Affiliations:** 1 Institut de Biologie Intégrative et des Systèmes (IBIS), Université Laval, 1030, avenue de la Médecine, Quebec, G1V 0A6 Canada; 2 Département de biochimie, de microbiologie et de bio-informatique, Faculté des sciences et de génie, Université Laval, 1045, avenue de la Médecine, Quebec, G1V 0A6 Canada; 3 Centre de recherche de l’Institut universitaire de cardiologie et de pneumologie de Québec, 2725, chemin Sainte-Foy, Quebec, G1V 4G5 Canada; 4 Department of Bacterial Genetics, Institute of Microbiology, Faculty of Biology, University of Warsaw, Ilii Miecznikowa 1, 02-096 Warsaw, Poland; 5 Groupe de Recherche en Écologie Buccale (GREB), Faculté de médecine dentaire, Université Laval, 2420, rue de la Terrasse, Quebec, G1V 0A6 Canada

**Keywords:** Insertion sequence, Plasmid, Homologous recombination, Virulence, *Aeromonas salmonicida*

## Abstract

**Background:**

*Aeromonas salmonicida subsp. salmonicida* is a ubiquitous psychrophilic waterborne bacterium and a fish pathogen. The numerous mobile elements, especially insertion sequences (IS), in its genome promote rearrangements that impact its phenotype. One of the main virulence factors of this bacterium, its type three secretion system (TTSS), is affected by these rearrangements. In *Aeromonas salmonicida subsp. salmonicida* most of the TTSS genes are encoded in a single locus on a large plasmid called pAsa5, and may be lost when the bacterium is cultivated at a higher temperature (25 °C), producing non-virulent mutants. In a previous study, pAsa5-rearranged strains that lacked the TTSS locus on pAsa5 were produced using parental strains, including 01-B526. Some of the generated deletions were explained by homologous recombination between ISs found on pAsa5, whereas the others remained unresolved. To investigate those rearrangements, short- and long-read high-throughput sequencing technologies were used on the *A. salmonicida* subsp. *salmonicida* 01-B526 whole genome.

**Results:**

Whole genome sequencing of the 01-B526 strain revealed that its pAsa5 has an additional IS copy, an IS*AS5*, compared to the reference strain (A449) sequence, which allowed for a previously unknown rearrangement to occur. It also appeared that 01-B526 bears a second large plasmid, named pAsa9, which shares 40 kbp of highly similar sequences with pAsa5. Following these discoveries, previously unexplained deletions were elucidated by genotyping. Furthermore, in one of the derived strains a fusion of pAsa5 and pAsa9, involving the newly discovered IS*AS5* copy, was observed.

**Conclusion:**

The loss of TTSS and hence virulence is explained by one consistent mechanism: IS-driven homologous recombination. The similarities between pAsa9 and pAsa5 also provide another example of genetic diversity driven by ISs.

**Electronic supplementary material:**

The online version of this article (doi:10.1186/s12864-017-3921-1) contains supplementary material, which is available to authorized users.

## Background

The bacterium *Aeromonas salmonicida subsp. salmonicida* is the causative agent of furunculosis, a disease that affects salmonids worldwide. It has a significant economic impact on the fish farming industry [1]. Vaccination and antibiotherapy are the available treatments for furunculosis. However, resistant *A. salmonicida* subsp. *salmonicida* strains have emerged [2–7] and vaccination is not always effective [8]. Alternative treatment options against this pathogen would thus be beneficial [8–10] but developing new treatments requires a better understanding of its underlying mechanisms, such as pathogenicity [4, 11].

This ubiquitous waterborne bacterium shows evidence of lateral gene transfer. *A. salmonicida* subsp. *salmonicida* strain genomes have been shown to bear a wide array of mobile elements [4, 12], genomic islands [13–15], and transferable plasmids [2, 3, 5, 16]. The close relationship between the mobile elements from this species and those of *Salmonella enterica* has raised concerns about its ability to act as a reservoir of antibiotic resistance genes, which could transfer to human pathogens [5, 7]. *Aeromonas salmonicida subsp. salmonicida* is also known for its genomic variability. Different kinds of recombination have formed plasmid and genomic island variants with altered gene contents that result in phenotypic changes [5, 6, 15–19]. This bacterium, beyond mediating gene transfer, could thus provide a new combination of those genes.

In many cases, insertion sequences (ISs) and transposons are responsible for these variations. They were found to participate in plasmid rearrangements, virulence factor inactivation, and the exchange of antibiotic resistance genes [16–20]. It was even suggested that the abundance of ISs (88 copies divided into 10 different types) in the reference genome A449 could maintain the bacterium in a psychrophilic lifestyle to preserve its genomic integrity [21]. Unfortunately, transposable elements also hinder genome sequencing and assembly, which are used to study structural variations [22–25].

One structural variation mediated by ISs that has been studied in *A. salmonicida* subsp. *salmonicida* is the loss of its type three secretion system (TTSS). This needle-like apparatus injects proteins named effectors into the host cells [26]. An interesting point for furunculosis management is the fact that the TTSS is essential for the pathogenicity of this bacterium [11, 27]. In *A. salmonicida* subsp. *salmonicida*, the TTSS structural components and many effectors are encoded on a plasmid called pAsa5 (also known as pASvirA) [12, 28], which has been found in many strains [12, 27, 28]. This 155-kilobase-pair (kbp) replicon harbours all TTSS structural genes in a single locus, four TTSS effector genes (*aopH*, *ati2*, *aopN*, and *aopO*), conjugative transfer genes, many uncharacterized open reading frames, and ISs [11, 12]. Interestingly, pAsa5 is a thermolabile plasmid that is known to lose segments, including the TTSS locus, when the cells are exposed to 25 °C and above [28, 29]. The resulting mutants are non-virulent in fish and in model hosts, making the TTSS loss mechanism an interesting target for avirulence-producing treatment development [27, 29–34].

In a previous study, a collection of TTSS-negative mutants were produced by prolonged cultivation at 25 °C of three *A. salmonicida* subsp. *salmonicida* parental strains: A449, 01-B526, and 01-B516 [29]. These plasmid-rearranged strains were shown to have two different types of truncated pAsa5.

**Fig. 1:**
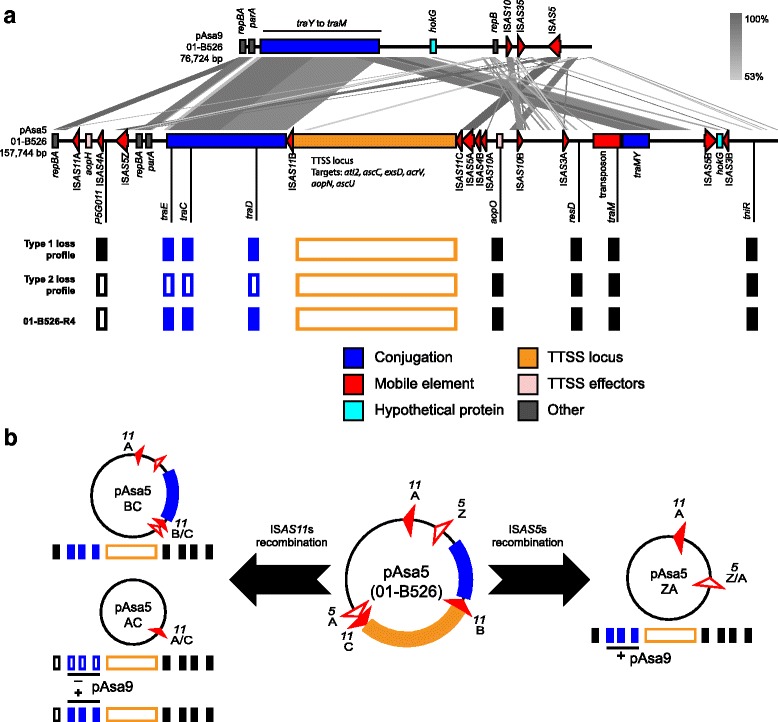
Alignment of 01-B526 pAsa5 and pAsa9, and proposed recombination patterns for 01-B526 pAsa5. **a** The plasmid sequences were aligned and visualized using EasyFig [52]. Featured ORFs and regions were coloured using the following code: *Red*: transposases or transposase fragments, *Blue*: conjugative transfer proteins, *Black*: ORFs with detailed functions or domains, *Cyan*: hypothetical protein, Orange: TTSS structural genes; *Light pink*: TTS effectors and chaperones. This alignment shows that shared regions between pAsa5 and pAsa9 are also syntenic. Moreover, pAsa9 has its conjugative transfer genes in a single locus, compared to two loci with a *traI* repetition for pAsa5. Below the alignment, PCR targets locations used by Daher et al. to assess pAsa5 integrity at stressful growth temperature, and the resulting loss profile, are shown [17, 29]. **b** IS locations on 01-B526 pAsa5 allow for two types of recombination patterns: IS*AS11*-targetted, which was described previously [17], and IS*AS5*-targetted. IS*AS11*B-IS*AS11*C recombination leads to pAsa5 BC, which has lost its TTSS locus (*top left circle*). IS*AS11*A-IS*AS11*C recombination leads to pAsa5 AC, which has lost its TTSS and conjugative transfer loci (*bottom left circle*). However, in 01-B526-R4, the conjugative locus is detected by PCR since a similar copy is carried by pAsa9 (bottom left rectangles). IS*AS5Z*-IS*AS5A* recombination leads to pAsa5 ZA, which has also lost its TTSS and conjugative transfer loci (*right circle*). Again, in this case the pAsa9 conjugative locus is detected by PCR, effectively masking the deletion

Plasmids displaying a type 1 deletion profile had lost their TTSS locus, which includes the essential structural proteins, effectors *ati2* and *aopN*, and their respective chaperones (Fig. 1a). Plasmids with a type 2 deletion profile lost their TTSS locus as well as 40 kbp upstream of it. Both deletion types led to complete virulence loss against an alternative host [29].

Further work revealed that all type 2 and some type 1 deletions could be explained by the same mechanism based on IS-mediated homologous recombination driven by the copies of IS*AS11* found on pAsa5 [17]. However, many strains that show a type 1 deletion profile could not be explained by the known IS recombination [17]. It was thus hypothesized that their plasmids had undergone incomplete or more complex recombinations.

As mentioned before, a better understanding of the genomic content and rearrangements in *A. salmonicida* subsp. *salmonicida* would be beneficial for virulence-targeted treatment development and for assessing the contributions of this species to gene flow by horizontal transfers. Different TTSS-loss mechanisms seem to occur in strains 01-B526 and A449. The genome of A449 has been already sequenced and fully assembled [12]. In this study, we combined Illumina and single molecule real-time (SMRT) sequencing for the 01-B526 genome to elucidate its plasmid composition. This helped us to identify another TTSS-loss scenario involved in unexplained pAsa5-rearranged strains.

## Results

### Characterization of pAsa9

**Fig. 2:**
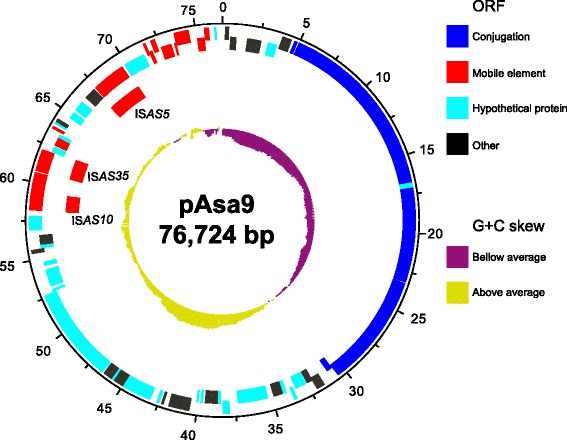
pAsa9 map. The pAsa9 sequence and features were visualized with Artemis and DNAPlotter [51, 54]. Going inwards from the scale (expressed in kbp), the first two circles mark ORFs (forward, then reverse strand) using the colour code introduced in Fig. 1. The third ring marks insertion sequences. The fourth marks GC skew, using the following colours: *purple*: below average; *yellow*: above average

A new plasmid sequence was obtained from the 01-B526 strain by combining short and long reads in a hybrid de novo assembly. The new plasmid, named pAsa9, is 76,724 base pairs with a G + C content of 52.76%, and 90 annotated open reading frames (ORF). It can be divided into sections according to its gene content and nucleotide similarities (Fig. 2). The first segment has replication-associated *repBA* genes (Fig. 2, 0 to 5 kbp) and has high nucleotide identity with two pAsa5 regions at 98.13% and 93.12%, respectively (Fig. 1a). The second region has many conjugation genes (*tra*, blue rectangles) and again was over 83% similar to pAsa5 (Fig. 1a). The third region, directly following *tra* genes, had many ORFs coding for hypothetical proteins (cyan, Fig. 2) or with less described functions (black, Fig. 2), but was still 84% similar (Fig. 1a) to pAsa5. Finally, pAsa9, the last region, was mostly filled with fragmented and complete transposase genes (Fig. 2, red rectangles).

Since ISs are a prominent component of *A. salmonicida* subsp. *salmonicida* genome [21], we investigated those of pAsa9. ISs and IS-related sequences were found solely in the last quarter of the sequence (Fig. 2, 57 kbp to end). More partial than complete ISs could be identified, suggesting ancestral transposition events [35]. Of the three complete ISs found on pAsa9, two, IS*AS10* and IS*AS5*, have already been identified and described in *A. salmonicida* subsp. *salmonicida* notably on the reference pAsa5 [12]. IS*AS35*, an IS that had not been described previously, was identified based on its transposase gene and inverted repeats homology. It belongs to the IS*Bst12* group, of which members have been found in *Klebsiella pneumoniae* (IS*Kpn15*, best score 248) and *A. salmonicida* subsp. *salmonicida* (IS*As21*, best score 167) based on the ISFinder database [36]. A blast analysis against deposited *Aeromonas* genomes (taxid:642, October 2016) showed IS*AS35* is only found in three *A. salmonicida* subsp. *salmonicida* genomes: 01-B526 (accession number AGVO01000000), J231 (LSGY01000000), and J227 (LSGX01000000).

### Comparison between pAsa9 and pAsa5

pAsa9 was sequenced from the strain 01-B526, which is already known to contain other plasmids: pAsa1, pAsa2, pAsa3, pAsal1 and pAsa5 [22]. Interestingly, pAsa9 shared long (up to 24 kbp), similar regions with pAsa5 (Fig. 1a). Shared similarities made it impossible to detect, assemble, and characterize pAsa9 in the previous studies focusing on 01-B526 genomics that were based on short-read sequencing only [22, 23]. High-throughput sequencing was sufficient to cover its specific regions since some contigs of *A. salmonicida* subsp. *salmonicida* J231 and J227 strains found in GenBank matched against pAsa9. However, the repeated regions between pAsa5 and pAsa9 did not allow for a complete assembly with previous 01-B526 datasets [22, 23]. SMRT and Illumina sequencing resulted in the assembly of both pAsa5 and pAsa9 as single contigs, which allowed us to distinguish them for the first time.

Apart from ISs, both plasmids share regions that encode conjugative transfer and plasmid replication genes. pAsa5 and pAsa9 have similar and contiguous set of *tra* genes (between 83% and 91%) and similar *repBA* genes (between 93% and 98%). Some regions, such as the *rep* surroundings and the *traI* gene, are repeated thrice between the plasmids: once on pAsa9 and twice on pAsa5. The distinct regions on pAsa9 compared to pAsa5 contained mostly hypothetical protein-coding sequences. On the other hand, pAsa5 unique regions carried hypothetical protein-coding sequences, but also ISs and, more importantly, TTSS-related genes. pAsa9 ISs and IS-associated genes are concentrated in the same region, while pAsa5 ISs are scattered throughout its length, and some are proximal to TTSS genes. For instance, the virulence-essential TTSS locus is framed by two IS*AS11*, as described earlier [17]. This region also disrupts the G + C skew in pAsa5 compared to pAsa9, suggesting a recent acquisition (Additional file 1). A 10 kbp region comprising another TTSS effector gene, *aopO,* is also framed by ISs, two IS*AS10*s. The third TTSS effector gene, *aopH*, is framed by non-identical ISs (IS*AS11* and IS*AS4*).

pAsa5 also shares similarities with pAsa6 (18.5 kbp), another *A. salmonicida* subsp. *salmonicida* plasmid that also has the *aopH* gene [37]. Sequence alignment showed that the genetic content and architecture of pAsa6 are closer to pAsa5 than pAsa9, since it carries the *aopH* section (Additional file 2). Furthermore, its similarities are concentrated around pAsa5’s first *rep* region and insertion sequences. The significant similarities between pAsa5 and pAsa9 suggest that pAsa6 has probably derived from pAsa5 through successive deletions instead of pAsa5 evolved from the fusion of pAsa6 with other plasmid(s).

### Genotyping unexplained type 1 loss profile and pAsa9 occurrence

01-B526 pAsa5 was aligned against the reference plasmid from the strain A449. The two plasmids had a high nucleotide identity (more than 99.5%) across their length. The only structural variation is an additional IS*AS5* inserted between the first IS*AS4* and the second *rep* region on 01-B526 pAsa5 (Fig. 1a). Since this IS copy is upstream from IS*AS5A* from the previous annotation [17], it was designated as IS*AS5Z* for the purpose of this paper. IS*AS5Z* (upstream from *tra* genes) and IS*AS5A* (downstream from the TTSS locus) are in the same orientation, hence they form a valid pair for IS-mediated loss by recombination.

This deletion would encompass both conjugation and TTSS loci, and should result in another loss profile for pAsa5 genotyping, a feature that had not been observed previously (Fig. 1b) [17, 29]. However, due to its similarity with pAsa9, primers hybridizing in the conjugation locus (*traE*, *traC*, and *traD*) can also hybridize on pAsa9, leading to false positives for this region (Fig. 1). IS*AS5Z*-IS*AS5A* recombination thus leads to a type 1 deletion profile in pAsa9-bearing strains when the previous genotyping primer set is used (Fig. 1b). The unexplained type 1 deletion profiles found in pAsa5-rearranged strains could thus be explained by IS*AS5*Z-A recombination, but only if the strains bore pAsa9 and an IS*AS5Z*.

All parental and derived strains were directly tested with primers flanking IS*AS5*Z 3′ and IS*AS5*A 5′ to assess the new ZA recombination. All parental strains and daughter strains for which a recombination pattern had already been found were negative to that new primer pair.

In the unexplained derived strains, all but one (01-B526-R19) were positive for the new ZA amplification, which gave a 2888 bp amplification product (Table 1). Sanger sequencing for that amplicon showed that it encloses an IS*AS5*, IS*AS5*Z flanking 3′, and IS*AS5*A flanking 5′, confirming the recombination. Those strains had thus experienced a homologous recombination between IS*AS5*Z and IS*AS5*A, leading to the loss of both TTSS and conjugation loci. All derived strains were then tested for the presence of pAsa9 using a set of genotyping primers that targeted its unique region (Additional file 3). A449-derived strains were negative for pAsa9, which was expected, since A449 does not carry this plasmid. All the other pAsa5-rearranged strains were positive for pAsa9. Since all newly assigned ZA pAsa5-bearing strains also carried pAsa9, it explained why the conjugation locus loss had not been detected in those strains previously [17].

**Table 1:** *A. salmonicida* subsp. *salmonicida* isolates used in this study

Isolates	Source	Previous A-C rearrangement genotyping	Previous B-C rearrangement genotyping	IS*AS5* Z-A rearrangement genotyping	pAsa9 genotyping
Parental strains					
A449	[32]	−	−	−	−
01-B526	[55]	−	−	−	+
01-B516	[29]	−	−	−	−
Displaying Type 1 Loss profile					
A449-R1	[29]	−	+	−	−
A449-R3	[29]	−	+	−	−
A449-R4	[29]	−	+	−	−
01-B526-R2	[29]	−	+	−	+
01-B526-R3	[29]	−	−	+	+
01-B526-R5	[29]	−	+	−	+
01-B526-R6	[29]	−	−	+	+
01-B526-R7	[29]	−	−	+	+
01-B526-R8	[29]	−	−	+	+
01-B526-R9	[29]	−	−	+	+
01-B526-R10	[29]	−	−	+	+
01-B526-R11	[29]	−	−	+	+
01-B526-R12	[29]	−	−	+	+
01-B526-R13	[29]	−	−	+	+
01-B526-R14	[29]	−	−	+	+
01-B526-R15	[29]	−	−	+	+
01-B526-R16	[29]	−	−	+	+
01-B526-R17	[29]	−	−	−	+
01-B526-R18	[29]	−	−	+	+
01-B526-R19	[29]	−	−	+	+
01-B526–3 (mistaken for 01-B516–3)	[29]	−	−	+	+
01-B526–11 (mistaken for 01-B516–11)	[29]	−	−	+	+
01-B526–30 (mistaken for 01-B516–30)	[29]	−	+	−	+
Displaying Type 2 loss profile					
A449-R2	[29]	+	−	−	−
A449-R5	[29]	+	−	−	−
01-B526-R4	[29]	+	−	−	+

### 01-B516 and its assigned daughter strains

The parental strains, A449, 01-B526 and 01-B516 were then tested for the presence of pAsa9. It was found that neither A449 (as expected) nor 01-B516 had the plasmid. However, all three 01-B516-derived strains (01-B516–3, 01-B516–11, and 01-B516–30) had pAsa9, creating a disparity between parental and offspring strains. It was thus hypothesized that during mutant production, some 01-B526-derived strains had been mistakenly labelled as 01-B516.

**Fig. 3:**
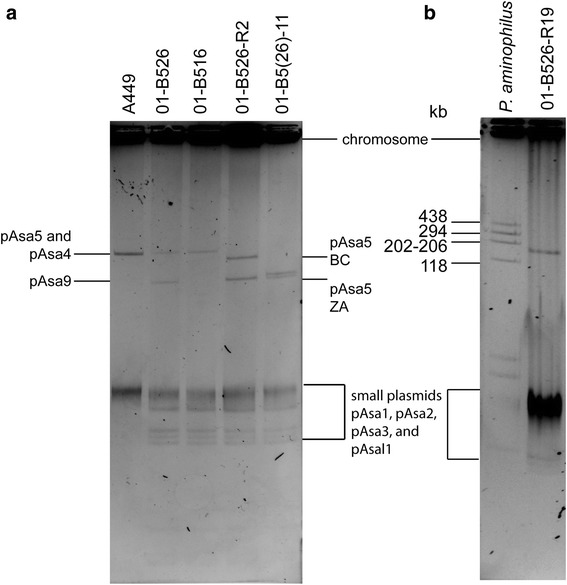
Large plasmids from parental and derived strains. A protocol adapted from Wheatcroft [53] was used to visualize large plasmids by agarose gel electrophoresis. **a** Some daughter strain profiles were compared to the parental strains. A449 displays a single band, corresponding to plasmids pAsa5 and pAsa4; 01-B526 displays pAsa5 and pAsa9 bands; and 01-B516 displays a single band, pAsa5. The 01-B526-R2 pAsa5 band is lower than 01-B526 one, since it underwent an IS*AS11*B-C recombination. 01-B516–11 displays a ZA-rearranged pAsa5 band as well as a pAsa9 one, whereas its parent does not have the pAsa9 plasmid. It was confirmed that all 01-B516-derived strains were mislabelled in the previous studies, and are in fact 01-B526-derived. **b** 01-B526-R19, the only derived strain whose deletion profile on pAsa5 cannot be explained by IS recombination, was also put on a gel beside *Paracoccus aminophilus* JCM 7686 [53]. The 01-B526-R19 single band has a molecular weight corresponding to its ZA-rearranged pAsa5 and pAsa9 fused together

To verify that 01-B516 did not have any form of pAsa9, plasmid profiles of all parental strains, one BC pAsa5-bearing strain (01-B526-R2), and one possibly mislabelled derived strain (01-B516–11), were visualized in agarose gel electrophoresis (Fig. 3a). As expected, A449 showed one band corresponding to pAsa5 and pAsa4 (155 kbp and 167 kbp, respectively). 01-B526 showed two bands, one for pAsa5 and one for pAsa9. 01-B516 showed a single large band whose weight matched pAsa5 (Fig. 3a). 01-B526-R2 showed the pAsa9 band as well as a BC-rearranged pAsa5 band. However, 01-B516–11, which was found to bear a ZA-rearranged pAsa5 according to our previous genotyping, gave two bands: one for the ZA pAsa5 and one whose size matched pAsa9. This result suggested this strain has a 01-B526-derived background and was thus mislabelled.

01-B516 genomic DNA was then sequenced using short-read, high-throughput sequencing (Illumina). When the resulting contigs were aligned on pAsa5 and pAsa9, it showed 01-B516 did not have sequences unique to pAsa9. Moreover, 01-B516 did not contain IS*AS5Z*, making an IS*AS5Z*-IS*AS5A* recombination impossible in this strain, nor in any daughter strain.

Derived strains 01-B516–3, 01-B516–11 and 01-B516–30 were also genotyped for *AsaGEI1a* (accession number KJ626178) presence and were found positive for that genomic island, whereas 01-B516 carries *AsaGEI2a* [13]. *AsaGEI*s (for *Aeromonas salmonicida* GEnomic Island) are found in some *A. salmonicida* subsp. *salmonicida* genomes. Each carrying strain only has one copy of one type of *AsaGEI*s. They have variable insertion sites (indicated by the number in their name) and gene content (indicated by their letter). They contain some phage-related genes and many ORFs coding for hypothetical protein. Consequently, their role remains to be determined [13–15].

Moreover, *AsaGEI* types are linked with their geographic origin and strains bearing *AsaGEI1a* are found in North America [13]. Since all 01-B516 ‘derived’ strains genotyped positive for pAsa9 and *AsaGEI1a*, and that two of them have a ZA-rearranged pAsa5, we concluded these three strains were mislabelled when they were obtained, and are instead 01-B526-derived strains.

### pAsa9 prevalence

The prevalence of pAsa9 in a collection of *A. salmonicida* subsp. *salmonicida* isolates was then determined by genotyping with primers covering its specific region (Additional file 3). Among the 154 genotyped strains, 35 had the plasmid and two generated amplicons using some primers only, suggesting another plasmid (Additional file 4). Furthermore, in our strain set, pAsa9 was always found with another element, the genomic island *AsaGEI1a*.

### Plasmid fusion in 01-B526-R19

One type 1 deletion profile strain, 01-B526-R19, could not be explained by any IS recombination, even with the ZA recombination discovery. 01-B526-R19 genomic DNA was thus sequenced by Illumina technology to detect potential new genomic content. Contig alignment to 01-B526 pAsa5 and pAsa9 showed it lacked the *tra* and TTSS regions on pAsa5, suggesting an IS*AS5*Z-A recombination, a fact which had been suspected but not proven by PCR. It also had a complete pAsa9, as shown by contig alignment.

The 01-B526-R19 recombination was deemed to be complex, so its plasmids were visualized on gel (Fig. 3b). 01-B526-R19 displayed a single large plasmid band at approximately 150 kbp, higher than a ZA-rearranged pAsa5 band, and no pAsa9 band was detected, even if its presence had been assessed by PCR and sequencing (Fig. 3b).

**Fig. 4:**
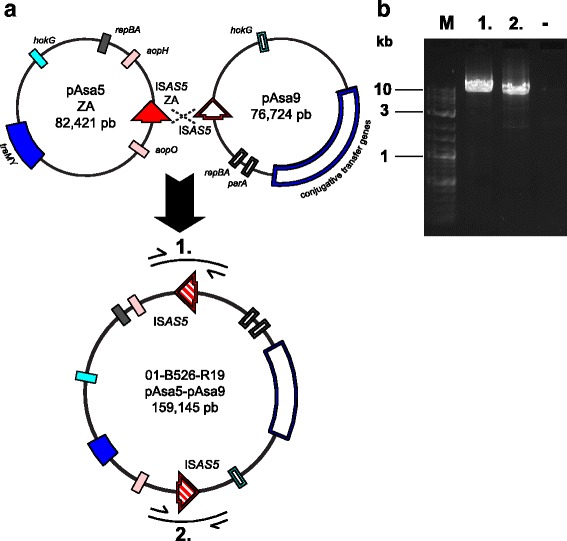
Fusion of the 01-B526-R19 pAsa5 plasmid with pAsa9 through their IS*AS5*. **a** The pAsa5 and pAsa9 fusion involved two IS*AS5* (full and hollow red arrows): IS*AS5Z*A, which is the product of a previous recombination, and pAsa9 sole IS*AS5*. Features from both plasmids that are found in Fig. 1 are included in the diagram for comprehension purposes, and ISs other than the involved IS*AS5s* were omitted for clarity. The figure is not to scale. The pAsa5 ZA-pAsa9 plasmid presence was visualized by gel electrophoresis (see Fig. 3b) and the IS*AS5* recombination and surrounding sequences were amplified by PCR (1 and 2). **b** Two long PCRs show the junctions between pAsa5 ZA and pAsa9. The numbers match the amplicon shown in the previous diagram. Product 1 is 9348 pb and Product 2 is 8429 pb. A negative control (−) targeting a TTSS region (Additional file 3) was also included

However, since the weight of the 01-B526-R19 band matched the sum of ZA-rearranged pAsa5 and pAsa9, and since both plasmids had one IS*AS5* copy, we hypothesized that both large plasmids had merged together by IS*AS5* recombination (Fig. 4a). Primers flanking IS*AS5*ZA in pAsa5 and pAsa9 IS*AS5* were used in a long amplification PCR (Additional file 4). Both sides of the merge were successfully amplified and confirmed by Sanger sequencing, indicating 01-B526-R19’s rearranged pAsa5 and its pAsa9 had indeed recombined into a single plasmid, and shedding light on the last unexplained strain recombination (Fig. 4b).

## Discussion

In this study, we used long-read sequencing (PacBio SMRT technology) to uncover a new *A. salmonicida* subsp. *salmonicida* plasmid, pAsa9. It would have been impossible to assemble pAsa9 with short-read sequencing information alone, due to its similarity with another well-studied, virulence-essential plasmid called pAsa5 [12, 29, 30]. Even with mate-pair sequencing, which allowed us, with a certain configuration, to assemble the *A. salmonicida* subsp. *salmonicida* chromosome in one scaffold [23], the pAsa9 assembly was complicated by its 24 kbp-long repeated sequence with pAsa5.

SMRT sequencing can be used to reduce contig numbers and manual gap filling for most microbial genomes, especially those that have many long repeats; this feature applies to *A. salmonicida* subsp. *salmonicida* [38]. Single-read sequencing was previously used to identify and assemble plasmids that shared a common backbone in the same strain [39]. PacBio or other long-read sequencing technologies should be used in future studies to facilitate the discovery of new plasmids in *A. salmonicida* subsp. *salmonicida*, especially if they are presumed to share repeats with the rest of the genome. Also, some species in the *Aeromonas* genus, such as *Aeromonas media* [40], have complex genomes and would thus benefit from that technology [21].

The newly discovered plasmid, pAsa9, was found in *A. salmonicida* subsp. *salmonicida* strains that also bear the genomic island *AsaGEI1a.* Thus, its presence is limited to North America so far based on the strains analysed. Interestingly, pAsa9 is found concurrently with pAsa5, even though their replication genes (*repBA*) are similar. Although the discovered plasmid, pAsa9, shares highly similar sequences with pAsa5, its unique content and architecture show that it is not a mere copy.

In fact, we propose that pAsa9 provides information on pAsa5 evolution and gene acquisition. The two plasmids are similar and have syntenic sequences, which suggests they share a common ancestor whose backbone should be closer to pAsa9 than pAsa5. Apart from its last quarter (Fig. 2), which encloses all mobile element-related sequences, pAsa9 is free from IS or sequence duplication. In contrast, pAsa5 has sequence duplications in core genes (replication and transfer) [41], and many ISs. Furthermore, some IS localizations follow TTSS genes.

We thus suggest that ISs were key elements in TTSS-related gene acquisition in pAsa5, which arose on a plasmid backbone close to pAsa9. The discovery of pAsa9 also places pAsa6, another pAsa5-related plasmid, as a pAsa5-like descendant rather than an ancestor, since its structure is closer to the latter (Additional file 2). Together, those results further prove that insertion sequences have a role in *A. salmonicida* subsp. *salmonicida* genetic diversity, as proposed earlier [21].

PacBio sequencing also provided new architectural details that allowed us to revisit previous experimental results on pAsa5 recombination and the associated loss of TTSS and virulence. With this information, all pAsa5 deletions that were generated in the study of Daher et al. can be regrouped under a consistent and simple mechanism, which is IS-mediated homologous recombination [29]. These results also allowed us to explain previous observations of pAsa5-rearranged strains that had lost their TTSS locus [17].

For instance, while attempting to produce TTSS-negative mutants, strain 01-B526 was more prone to producing plasmid-rearranged strains than strain A449 [29]. This can be explained by the fact that in 01-B526 pAsa5, another IS (IS*AS5*Z) can be a target for recombination.

With 2.6 kbp, IS*AS5* offers a large span of homologous sequences for recombination to occur, which could explain why IS*AS5* rearrangements were observed more frequently in 01-B526-derived strains than IS*AS11* rearrangements. IS*AS5* was already known as an active insertion sequence in *A. salmonicida* subsp. *salmonicida*. It was found to have transposed at multiple locations on pAsal1 [18, 19]. The results thus suggest that IS*AS5* is both an insertion sequence capable of transposition, and a target for host-mediated recombination. 01-B526 also gave only one IS*AS11*A-C recombinant with an atypical genotyping profile, 01-B526-R4, which can now be explained by the presence of pAsa9 and the false positive *tra* targets it creates (Fig. 1). Thus, PacBio sequencing both provided an explanation for shaded areas left by the previous study, and simplified some cases.

One exception is 01-B526-R19, which has a fusion between its ZA-rearranged pAsa5 and its pAsa9. This merged plasmid is a rarity since it resulted from two sequential recombinations, one between IS*AS5*Z and *5*A, and one with IS*AS5*ZA and pAsa9 IS*AS5*. Such double recombinations between IS*AS11*s were tested in 2012 with no success [17]. This may be due to the fact, as mentioned earlier, IS*AS11* is half the size of IS*AS5*.

The reassignment of derived strains 01-B516–3, 01-B516–11 and 01-B516–30 to 01-B526 leaves 01-B516 without pAsa5-rearranged descendants that would prove the strain is capable of IS-targeted recombination. Difficulties in producing 01-B516 pAsa5-rearranged strains were observed in the original experiment [29]. This problem could be caused either by the structure of 01-B516 pAsa5 or by the lack of suitable recombinase. The Illumina sequencing showed that IS*AS11*B and IS*AS11*C are in the correct position relative to each other to mediate the deletion. BC recombination and TTSS loss should thus have been observed. Consequently, the lack of rearrangement in 01-B516 may be due to a missing or inactive recombinase.

Furunculosis management needs new alternative treatments to antibiotherapy and vaccination. Avirulent treatment suppresses essential pathogenicity mechanisms without killing the bacteria, and could be an option against *A. salmonicida* subsp. *salmonicida*. However, full understanding of the TTSS loss and the underlying IS-mediated recombination is a prerequisite to finding new ways to attenuate furunculosis-causing strains. Now, factors promoting these events can be investigated in the hopes of finding a specific, aquaculture-compatible condition that would trigger recombination of pAsa5.

## Conclusions

Our results showed that PacBio sequencing could provide crucial information for plasmid architecture studies by allowing for proper separation of long repeated regions. In this case, it led to the discovery of a new plasmid, pAsa9, which in turn allowed refinement and completion of the recombination model described for pAsa5 in 2012 [17].

Regrouping all TTSS-loss scenarios under a simple rule was an important step in investigating the mechanism for avirulence-producing treatments. The relationship between the pAsa9 and pAsa5 architectures demonstrates the importance of ISs in producing biologically-relevant genomic diversity in *A. salmonicida* subsp. *salmonicida*.

## Methods

### Bacterial isolates and growth conditions

The *A. salmonicida* subsp. *salmonicida* strains that were used in this study and are listed in Table 1 (strains listed in Tanaka et al.) and Additional file 4 (all other strains, used for genotyping). In Table 1, pAsa5-rearranged strains (that display type 1 or type 2 loss profiles) were obtained by culturing virulent parental strains (A449, 01-B516 and 01-B526) for 2 weeks at 25 °C [29]. All *A. salmonicida* strains were grown on furunculosis agar (10 g of tryptone, 5 g of yeast extract, 1 g of L-tyrosine, 2.5 g of NaCl, and 15 g of agar per litre of distilled water) for 2 or 3 days at 18 °C [42]. *Paracoccus aminophilus* JCM 7686 was used as a DNA size marker in plasmid visualization [43]. It was grown on tryptic soy agar (EMD Millipore, Ontario, Canada) for 3 days at 30 °C.

### DNA extraction and sequencing

Total genomic DNA was extracted for strains 01-B526 and 01-B516, and derived strain 01-B526-R19 (Table 1) using DNeasy Blood and Tissue kits (Qiagen, Canada). Libraries were prepared with a KAPA Hyper Prep Kit and sequencing was done at the Plateforme d’Analyse Génomique of the Institut de Biologie Intégrative et des Systèmes (IBIS, Université Laval, Quebec City, Canada) using the Illumina MiSeq system. Strain 01-B526 total genomic DNA was also extracted by phenol/chloroform by following the protocol *Extracting DNA Using Phenol-Chloroform* provided by Pacific Biosciences (http://www.pacb.com) and was sequenced at the Génome Québec Innovation Centre (McGill University, Montreal, Canada) using a Pacific BioScience RS II system. A hybrid de novo assembly was used for 01-B526 reads using Spades version 3.6.0 (kmer lengths of 21, 33, 55, 77, 99, 127) [44]. 01-B516 and 01-B526-R19 reads were assembled using A5-miseq version 20150522 [45].

### Sequence analysis

The 01-B526 assembly allowed us to obtain the large plasmids as complete closed sequences. pAsa5 and pAsa9 were annotated using the webserver RAST (February 2016) [46]. Manual verification was done using Artemis version 16.0.0 using pAsa5 reference sequence (NC_009350) for comparison and blastp (non-redundant protein sequences database, May 2016) [47, 48]. Manual verification of the mobile genetic elements was done with ISFinder assistance (June 2016) [36]. For 01-B516 and 01-B526-R19, contigs were aligned on 01-B526 pAsa5 and pAsa9 sequences using CONTIGuator version 2.7.4 [49]. Sequence alignment visualization was done with ACT version 13.0.0, DNA Plotter version 10.2 and EasyFig version 2.1 [50–52].

### PCR analyses

For PCR, amplifications up to 3 kbp were performed as previously described [18], with the exception of the elongation time, which was 1 min per 1 kbp of amplicon. For amplicons longer than 3 kbp, each reaction was built as follows: 3.75 μL of 2 mM dNTPs, 1 μL of each 100 ng/μL forward and reverse primers, 1 μL of 100 ng/μL template, 12.25 μL of H_2_O, 5 μL of 5X LongAmp Taq buffer (New England BioLabs, Massachusetts, US), and 1 μL LongAmp Taq (New England BioLabs). For those amplicons, the elongation time was 50 s per 1 kb of amplicon. New PCR primers were designed manually and verified with Oligoanalyser 3.1 from Integrated DNA Technologies (IDT, http://www.idtdna.com/calc/analyzer). The PCR assays were performed at least twice, and appropriate positive and negative controls were included with each assay. The PCR primers are listed in Additional file 3.

### Plasmid visualization by agarose gel electrophoresis

*A. salmonicida* subsp. *salmonicida* plasmids were visualized by agarose gel electrophoresis using a protocol adapted from Wheatcroft et al. [53]. 160 μL of overnight *P. aminophilus* JCM 7686 in tryptic soy broth (EMD Millipore) or *A. salmonicida* subsp. *salmonicida* in lysogeny broth (EMD Millipore) were mixed with 1 mL ice-cold 2% N-lauroyl sarcosine and centrifuged at 17200 x *g* for 10 min at 4 °C. Supernatants were discarded and pellets were suspended in 40 μL 10 mM Tris, 10 mM EDTA, 4 mg/mL RNAse, 1 mg/mL lysozyme, 1 mg/mL bromophenol blue and 15.2% Ficoll solution. Samples were incubated on ice for 45 min. A 0.75% agarose gel in Tris-borate-EDTA buffer (TBE 1X) was loaded with 40 μL of SDS 10%, 0.5% xylene cyanol solution, which was migrated backwards at 100 V for 15 min. DNA samples were then centrifuged at 376 x *g* for 20 s, then 20 μL were loaded on the gel. The polarity was inverted back and samples were migrated 30 min at 40 V, and then 5–7 h at 100 V. Gels were stained in 1 μg/mL ethidium bromide for 40 min, rinsed in water for 1 h to remove the excess of ethidium bromide, and photographed using short-length UV transilluminator.

## Additional files

Additional file 1: Circular map of pAsa5 of 01-B526 strain with G + C skew. pAsa5 sequence and features were visualized with Artemis and DNAPlotter [51]. From the outermost ring moving inwards, the first two circles shows open reading frames (forward, then reverse strand) in the colours described in Fig. 1. The third circle shows mobile elements. The fourth circle shows the G + C skew, using the following colours: purple: below average; yellow: above average. (PDF 171 kb)

Additional file 2: Alignment between pAsa5 and pAsa9 from 01-B526 with pAsa6. Methods and colour shown are the same as Fig. 1. (PDF 165 kb)

Additional file 3: Primers used in this study. This table lists the primers used for pAsa5, pAsa9 and *AsaGEI1a* genotyping, IS recombination detection, plasmid fusion detection and positive control. (DOCX 66 kb)

Additional file 4: Strains genotyped in this study. These strains were genotyped for the presence of pAsa9 and *AsaGEI1a. (DOCX 255 kb)*

## Abbreviations

IS: Insertion sequence
ORF: Open reading frame
SMRT: Single molecule real-time
TTSS: Type three secretion system
