# Supplementary figures and images for: Plasmid composition in Aeromonas salmonicida subsp. salmonicida 01-B526 unravels unsuspected type three secretion system loss patterns
Source: BMC Genomics. 2017 Jul 12;18:528. doi: 10.1186/s12864-017-3921-1 (PMC5508783; doi:10.1186/s12864-017-3921-1)

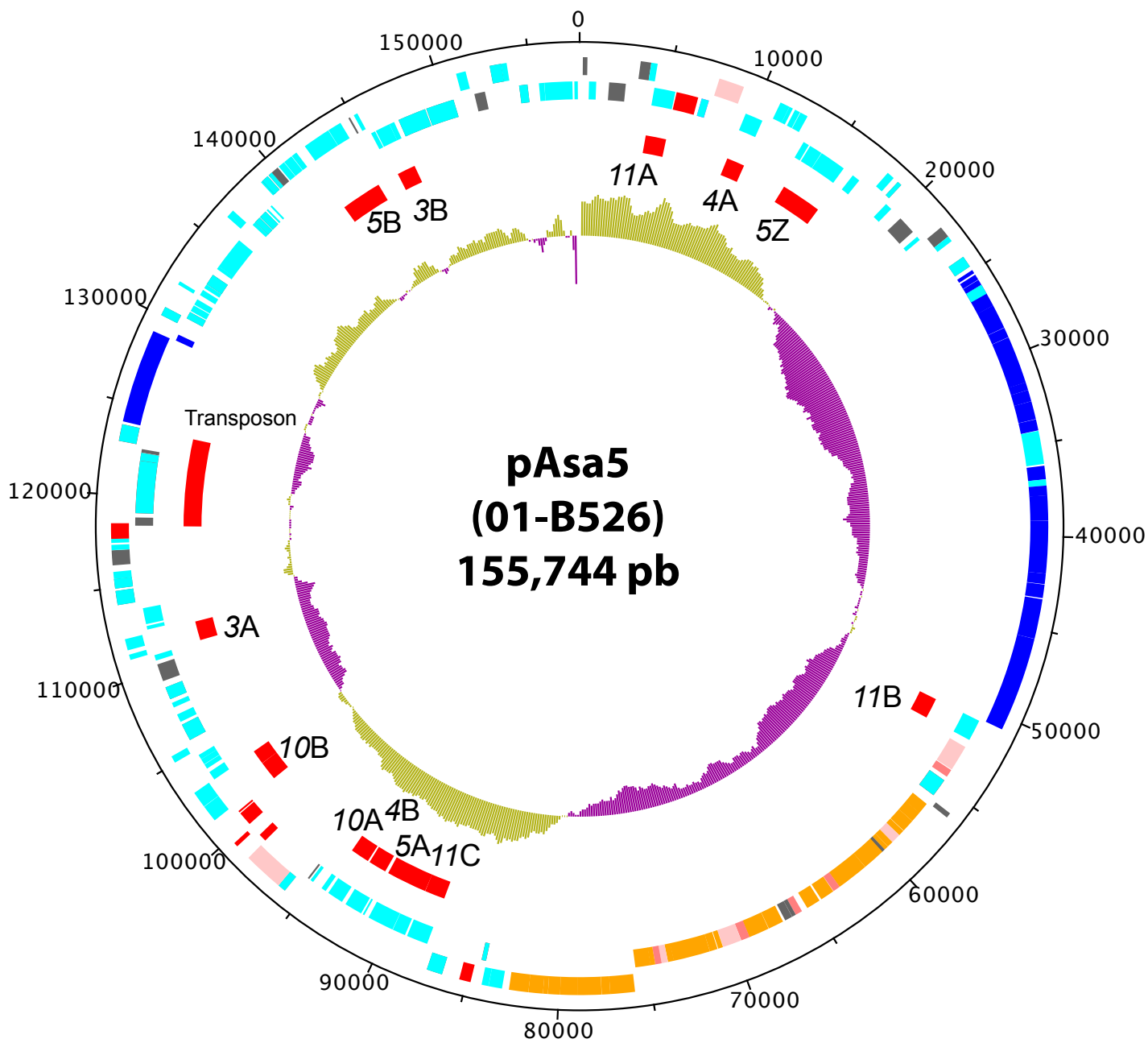

Supplement: Supplementary file 1 — Circular map of pAsa5 of 01-B526 strain with G + C skew. pAsa5 sequence and features were visualized with Artemis and DNAPlotter [51]. From the outermost ring moving inwards, the first two circles shows open reading frames (forward, then reverse strand) in the colours described in Fig. 1. The third circle shows mobile elements. The fourth circle shows the G + C skew, using the following colours: purple: below average; yellow: above average. (PDF 171 kb) [file 12864_2017_3921_MOESM1_ESM.pdf]

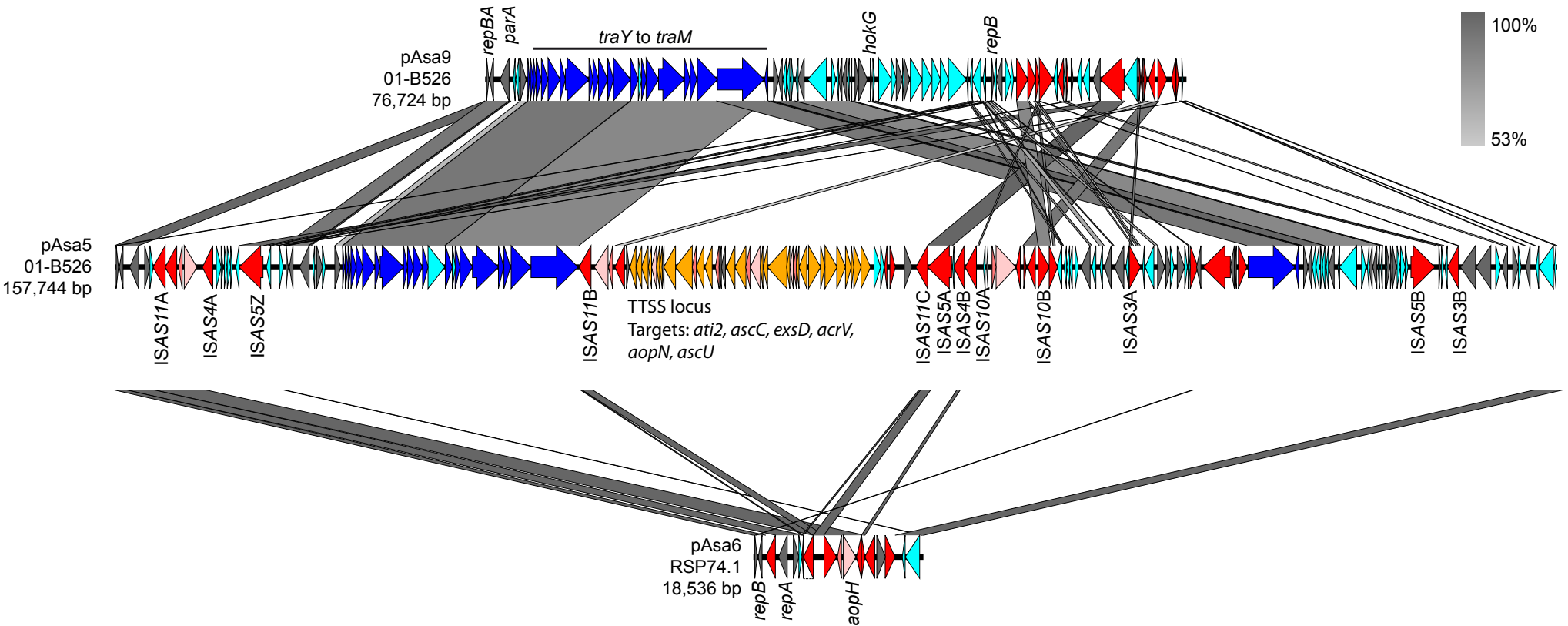

Supplement: Supplementary file 2 — Alignment between pAsa5 and pAsa9 from 01-B526 with pAsa6. Methods and colour shown are the same as Fig. 1. (PDF 165 kb) [file 12864_2017_3921_MOESM2_ESM.pdf]
