# Supplementary material for: Plasmid composition in Aeromonas salmonicida subsp. salmonicida 01-B526 unravels unsuspected type three secretion system loss patterns
Source: BMC Genomics. 2017 Jul 12;18:528. doi: 10.1186/s12864-017-3921-1 (PMC5508783; doi:10.1186/s12864-017-3921-1)
Supplement: Supplementary file 3 — Primers used in this study. This table lists the primers used for pAsa5, pAsa9 and AsaGEI1a genotyping, IS recombination detection, plasmid fusion detection and positive control. (DOCX 66 kb) [file 12864_2017_3921_MOESM3_ESM.docx]

**Additional file 3.** **Primers used in this study**

| Pair | Primer | Sequence (5’ 🡪 3’) | Reference |
| --- | --- | --- | --- |
| pAsa5 genotyping | | | |
| a | P5G011-Fwd* | TTGTCTGACTCTGCATCCAGCGAA | [[1](#_ENREF_1)] |
|  | P5G011-Rev | AGGTGCCTGAATTACCACCAGTGA | [[1](#_ENREF_1)] |
| b | *traE*-Fwd | ATGGTCGCTATCGTCATTGTCGCA | [[1](#_ENREF_1)] |
|  | *traE*-Rev | ACCTCGTTGCGCTTTATTTGCTGG | [[1](#_ENREF_1)] |
| c | *traC*-Fwd | TGCACTATCCCCAGCTATCC | [[1](#_ENREF_1)] |
|  | *traC*-Rev | TCGGTAATCGCGGTCTTGTC | [[1](#_ENREF_1)] |
| d | *traD*-Fwd | ATTCATGACCAATCCGCCGACCTA | [[1](#_ENREF_1)] |
|  | *traD*-Rev | TCGAGAAATTTGCGCATGAGCGTC | [[1](#_ENREF_1)] |
| e | *ati2*-Fwd‡ | TTGACCTGTGGTCAGGTTAGCAGT | [[1](#_ENREF_1)] |
|  | *ati2*-Rev | ACACGATGATACGCACCTAGCCAA | [[1](#_ENREF_1)] |
| f | *ascC*-Fwd‡ | GCATTGGAGCAACAGTCCCA | [[1](#_ENREF_1)] |
|  | *ascC*-Rev | CCTTCAATCCCCTTGCGAT | [[1](#_ENREF_1)] |
| g | *exsD*-Fwd | AGAAGTGATCCTGACCCAAGGCAA | [[1](#_ENREF_1)] |
|  | *exsD*-Rev | TTGCAACGACTGTTGCCAAGAACC | [[1](#_ENREF_1)] |
| h | *acrV*-Fwd | GTAAAGGGTTGCGGGATGAG | [[1](#_ENREF_1)] |
|  | *acrV*-Rev | GCCGCTCTTCTTCAGGTCAC | [[1](#_ENREF_1)] |
| i | *aopN*-Fwd | GCATCAGCGAGATCGAGG | [[1](#_ENREF_1)] |
|  | *aopN*-Rev | CGGCTGTGAATATCGC | [[1](#_ENREF_1)] |
| j | *ascU*-Fwd | GCTGGTCATCTACATCAAGC | [[1](#_ENREF_1)] |
|  | *ascU*-Rev | TAGTGTTCGAAGGCGTAGTC | [[1](#_ENREF_1)] |
| k | *aopO*-Fwd | TCCACAACGACATCAAACCGGGTA | [[1](#_ENREF_1)] |
|  | *aopO*-Rev | TGATCTGACCCTGATCGGCAAACT | [[1](#_ENREF_1)] |
| l | *resD*-Fwd | TCAGAAACTTGGCCATCGCTCACA | [[1](#_ENREF_1)] |
|  | *resD*-Rev | TCAACGTCCCAGTAACAGCGGATT | [[1](#_ENREF_1)] |
| m | *traM*-Fwd | AAGGTAATCGGAAGCACCTGCCAT | [[1](#_ENREF_1)] |
|  | *traM*-Rev | TTCTCGACACCAACGTGTTGCTCA | [[1](#_ENREF_1)] |
| n | *tniR*-Fwd | TGACACAGATCACCGACCTTGGTT | [[1](#_ENREF_1)] |
|  | *tniR*-Rev | TGGCATTGGCCTCAAAGTGTTGAC | [[1](#_ENREF_1)] |
| o | *tral*-Fwd | GTGGCAAATGAACGAGCCGAGTTT | [[1](#_ENREF_1)] |
|  | *tral*-Rev | TGCCAACTTACGCAATTGTCGCTC | [[1](#_ENREF_1)] |
| pAsa9 genotyping | | | |
| p | Orf6-Fwd | GGGATCCACCGCAGCAGTTATAAA | This work |
|  | Orf6-Rev* | GGGCCTTCATCGTCAACTCACTAT | This work |
| q | Orf19-Fwd | GCCGTCTCCACCAAGGACAGTATT | This work |
|  | Orf19-Rev† | CGTAATGTGGCTGCCATCGGACAA | This work |
| r | Orf25-Fwd | GCCGAACGCCAGTTCCAAATTGAT | This work |
|  | Orf25-Rev | CGCCTCAATTTGTGACCATGACCA | This work |
| s | Orf32-Fwd | CGAGAGAATGAGGCAGTACGGCAA | This work |
|  | Orf32-Rev | CGGTATGGCAAAGTCCTCTTCTGA | This work |
| t | Orf40-Fwd | CGCACTTGCTGTTAGACGCATGAA | This work |
|  | Orf40-Rev | GGCAGACCAAGAGCTTGTGCAGTT | This work |
| u | Orf44-Fwd | CCTGCGCGACGGCAGAAATAAACA | This work |
|  | Orf44-Rev | GGCTCAAAAGGCATTTGGCACGAT | This work |
| v | Orf49-Fwd | CTGCATTGAACACTCGGCTTCCTA | This work |
|  | Orf49-Rev | CCGTGTTGAAGCACGAATCAAACG | This work |
| Recombination genotyping | | | |
| w | 11A-Fwd | AATAGGTGTCGCAAGCTGGGTTGA | [[2](#_ENREF_2)] |
| x | 11B1-Fwd | GCGCACCACCACCATTTAATGTCA | [[2](#_ENREF_2)] |
| w-x | 11C-Rev | AACTGGCAAGGATAGAGCTGCTGA | [[2](#_ENREF_2)] |
| y | 5Z-Fwd | TCAGTGCCAATCAAATCAAACTCC | This work |
|  | 5A2-Rev† | TCCACGACAAACTGAATAAACTGG | This work |
| *AsaGEI1a* genotyping | | | |
| z | ins1-F1 | AAATACCTTGTGGCGGGCAAAGTC | [[3](#_ENREF_3)] |
|  | ins1-R1 | ATTTGACATCGCACGGAAACCTCG | [[3](#_ENREF_3)] |
| aa | orf11-F1 | GCGCTGGCCAACATATTCATCAGT | [[3](#_ENREF_3)] |
|  | orf11-R2 | AGCTGTTTAGCCACCCAGGTATGT | [[3](#_ENREF_3)] |
| bb | orf19-F1 | TGAACTTCGTCACCGACGACAACA | [[3](#_ENREF_3)] |
|  | orf19-R1 | TCAATCAGAATTGCCATTGGCGGG | [[3](#_ENREF_3)] |
| cc | orf33-F2 | ATGTGCTGTTGTTGGCCGTACTTG | [[3](#_ENREF_3)] |
|  | orf33-R2 | GTTTGGTCTGAAATGTGAGCGCGA | [[3](#_ENREF_3)] |
| dd | orf45-F2 | ACATGGCGTTGGGATTAACGATGC | [[3](#_ENREF_3)] |
|  | orf45-R2 | ACAGGGATGAAGGCACAGGTTGTA | [[3](#_ENREF_3)] |
| ee | orf54-F2 | TGCCTATGTCGAACTCTCCCACAA | This work |
|  | orf54-R2 | CCTTGCACTTCGCATTGAGGC | This work |
| ff | orf55-F1 | AAGAACAAATCGCCCAAGCGCAAG | This work |
|  | orf55-R1 | AGTTATAGGCTCGCTCACAGGCTA | This work |
| gg | orf57-F1 | TGCGTCATGTTCATGCTGGATGTG | This work |
|  | orf57-R1 | ATCCGGTGAGTTGGTGTCCATGAT | This work |
| hh | orf58-F1 | GTCCATGCACATCGACAATGCCAA | This work |
|  | orf58-R1 | TCTTGACCTCGGCCATGAACTTCT | This work |
| ii | orf64-F1 | CCAAGAACAAGGGTCAAACCGTCA | This work |
|  | orf64-R2 | ACGAAGCGGAACTCTTCCACAGAT | This work |
| jj | orf66-F2 | ATCCAGCTGCTGCGTAATGATTGG | This work |
|  | orf66-R2 | GCTTCACCAGCAACCCGTAATAGA | This work |
| kk | orf67-F2 | CTGACGCATCTTGCTGTCACCATT | [[3](#_ENREF_3)] |
|  | orf67-R1 | ATCGGTGCATTGGTGTACCCGTAA | [[3](#_ENREF_3)] |
| ll | orf72-F2 | GCGATCAGCTGCTGCATAAGAACA | This work |
|  | orf72-R1 | ATGCGGACGGGAACTTAGTCCAAT | This work |
| mm | orf78-F2 | AAGACGGTCTGGAACTTGGCTGAT | This work |
|  | orf78-R1 | CGTTGCCATTGACATAGCCACCAA | This work |
| nn | orf80-F2 | GCCGCTATTGAACTACTCCCAGTT | This work |
|  | orf80-R2 | TACCGGATGCTGAACCAGGTGAAT | This work |
| oo | ins2-F1 | GTTCGGCAGGTGGAATGTTGGTTT | [[3](#_ENREF_3)] |
|  | ins2-R1 | AGCGTTCACCCATATTCTCCCACT | [[3](#_ENREF_3)] |
| Chromosomal control | | | |
| pp | *tapA*-Fwd | ACATGAAGAAGCAATCAGGC | [[4](#_ENREF_4)] |
|  | *tapA*-Rev | AGAGGTCATGCGTTAGCAG | [[4](#_ENREF_4)] |

*, †, and ‡ primers were used together in long amplification.

References

1. Daher RK, Filion G, Tan SG, Dallaire-Dufresne S, Paquet VE, Charette SJ: Alteration of virulence factors and rearrangement of pAsa5 plasmid caused by the growth of *Aeromonas salmonicida* in stressful conditions. Vet Microbiol. 2011;152(3-4):353-360.

2. Tanaka KH, Dallaire-Dufresne S, Daher RK, Frenette M, Charette SJ: An insertion sequence-dependent plasmid rearrangement in *Aeromonas salmonicida* causes the loss of the type three secretion system. PloS one. 2012;7(3):e33725.

3. Emond-Rheault JG, Vincent AT, Trudel MV, Brochu F, Boyle B, Tanaka KH et al: Variants of a genomic island in *Aeromonas salmonicida* subsp. *salmonicida* link isolates with their geographical origins. Vet Microbiol. 2015;175(1):68-76.

4. Ebanks RO, Knickle LC, Goguen M, Boyd JM, Pinto DM, Reith M et al: Expression of and secretion through the *Aeromonas salmonicida* type III secretion system. Microbiology. 2006;152(Pt 5):1275-1286.
