# Supplementary material for: Plasmid composition in Aeromonas salmonicida subsp. salmonicida 01-B526 unravels unsuspected type three secretion system loss patterns
Source: BMC Genomics. 2017 Jul 12;18:528. doi: 10.1186/s12864-017-3921-1 (PMC5508783; doi:10.1186/s12864-017-3921-1)
Supplement: Supplementary file 4 — Strains genotyped in this study. These strains were genotyped for the presence of pAsa9 and AsaGEI1a. (DOCX 255 kb) [file 12864_2017_3921_MOESM4_ESM.docx]

Additional file 4 : Strains genotyped in this study

| Strain | Origin‡ | Fish‡ | *AsaGEI1a* | pAsa9 | Reference |
| --- | --- | --- | --- | --- | --- |
| 01-B522 | Quebec, Canada | Brook trout | + [[1](#_ENREF_1)] | + | [[2](#_ENREF_2)] |
| 01-B526 | Quebec, Canada | Brook trout | + [[1](#_ENREF_1)] | + | [[3](#_ENREF_3)] |
| 01-B516 | Quebec, Canada | Brook trout | - [[1](#_ENREF_1)] | - | [[2](#_ENREF_2)] |
| HER1098 | United States | INA | - [[1](#_ENREF_1)] | - | [[2](#_ENREF_2)] |
| HER1110 | Japan | INA | - [[1](#_ENREF_1)] | - | [[4](#_ENREF_4)] |
| HER1108 | Denmark | INA | - [[1](#_ENREF_1)] | - | [[4](#_ENREF_4)] |
| HER1104 | France | INA | - [[1](#_ENREF_1)] | - | [[2](#_ENREF_2)] |
| HER1085 | Norway | Trout | - [[1](#_ENREF_1)] | - | [[2](#_ENREF_2)] |
| HER1084 | France | INA | - [[1](#_ENREF_1)] | - | [[4](#_ENREF_4)] |
| HER1107 | INA | INA | - [[1](#_ENREF_1)] | - | [[2](#_ENREF_2)] |
| A449 | France | Brown trout | - [[1](#_ENREF_1)] | - | [[5](#_ENREF_5)] |
| 07-9324 | Quebec, Canada | Brook trout | + [[1](#_ENREF_1)] | + | [[2](#_ENREF_2)] |
| 07-7817 | Quebec, Canada | INA | + [[1](#_ENREF_1)] | + | [[2](#_ENREF_2)] |
| 07-7346 | Quebec, Canada | Atlantic salmon | - [[1](#_ENREF_1)] | - | [[2](#_ENREF_2)] |
| 07-5957 | Quebec, Canada | Atlantic salmon | - [[1](#_ENREF_1)] | - | [[2](#_ENREF_2)] |
| 08-2647 | Quebec, Canada | Brook trout | + [[1](#_ENREF_1)] | + | [[2](#_ENREF_2)] |
| 09-0167 | Quebec, Canada | Atlantic salmon | - [[1](#_ENREF_1)] | - | [[2](#_ENREF_2)] |
| 07-7287 | Quebec, Canada | Brook trout | + [[1](#_ENREF_1)] | + | [[2](#_ENREF_2)] |
| 08-2783 | Quebec, Canada | Brook trout | + [[1](#_ENREF_1)] | + | [[2](#_ENREF_2)] |
| 08-4188 | Quebec, Canada | Brook trout | + [[1](#_ENREF_1)] | + | [[2](#_ENREF_2)] |
| m17524-09 | Quebec, Canada | Brook trout | - [[1](#_ENREF_1)] | - | [[6](#_ENREF_6)] |
| m14349-09 | Quebec, Canada | Atlantic salmon | + [[1](#_ENREF_1)] | + | [[6](#_ENREF_6)] |
| m23281-09 | Quebec, Canada | Brook trout | - [[1](#_ENREF_1)] | - | [[6](#_ENREF_6)] |
| m23067-09 | Quebec, Canada | Brook trout | - [[1](#_ENREF_1)] | - | [[6](#_ENREF_6)] |
| m19438-09 | Quebec, Canada | Brook trout | - [[1](#_ENREF_1)] | - | [[6](#_ENREF_6)] |
| m16583-09 | Quebec, Canada | Brook trout | - [[1](#_ENREF_1)] | - | [[6](#_ENREF_6)] |
| m14231-09 | Quebec, Canada | Atlantic salmon | - [[1](#_ENREF_1)] | - | [[6](#_ENREF_6)] |
| m11743-09 | Quebec, Canada | Brook trout | - [[1](#_ENREF_1)] | - | [[6](#_ENREF_6)] |
| m11431-09 | Quebec, Canada | Brook trout | - [[1](#_ENREF_1)] | - | [[6](#_ENREF_6)] |
| m10419-09 | Quebec, Canada | Brook trout | - [[1](#_ENREF_1)] | - | [[6](#_ENREF_6)] |
| m9906-09 | Quebec, Canada | Brook trout | + [[1](#_ENREF_1)] | + | [[6](#_ENREF_6)] |
| m9954-10 | Quebec, Canada | Brook trout | + [[1](#_ENREF_1)] | + | [[6](#_ENREF_6)] |
| m8029-10 | Quebec, Canada | Brook trout | - [[1](#_ENREF_1)] | - | [[6](#_ENREF_6)] |
| m11603-10 | Quebec, Canada | Brook trout | - [[1](#_ENREF_1)] | - | [[6](#_ENREF_6)] |
| m6363-10 | Quebec, Canada | Brook trout | - [[1](#_ENREF_1)] | - | [[6](#_ENREF_6)] |
| m9221-10 | Quebec, Canada | Brook trout | - [[1](#_ENREF_1)] | - | [[6](#_ENREF_6)] |
| 2009-178 K9 | New Brunswick, Canada | Atlantic salmon | - [[1](#_ENREF_1)] | - | [[6](#_ENREF_6)] |
| 2009-157 K5 | New Brunswick, Canada | Brook trout | - [[1](#_ENREF_1)] | - | [[6](#_ENREF_6)] |
| 2010-47 K18 | New Brunswick, Canada | Brook trout | - [[1](#_ENREF_1)] | - | [[6](#_ENREF_6)] |
| 2004-05 MF26 | New Brunswick, Canada | INA | - [[1](#_ENREF_1)] | - | [[6](#_ENREF_6)] |
| 2004-68 K52 | New Brunswick, Canada | Atlantic salmon | - [[1](#_ENREF_1)] | - | [[6](#_ENREF_6)] |
| 2009-195 K29 | New Brunswick, Canada | Brook trout | - [[1](#_ENREF_1)] | - | [[6](#_ENREF_6)] |
| 2005-70 | New Brunswick, Canada | INA | - [[1](#_ENREF_1)] | - | [[6](#_ENREF_6)] |
| 2009-144 K3 | New Brunswick, Canada | Brook trout | - [[1](#_ENREF_1)] | - | [[6](#_ENREF_6)] |
| 2005-175 K2 | New Brunswick, Canada | Brook trout | - [[1](#_ENREF_1)] | - | [[6](#_ENREF_6)] |
| 2004-208 | New Brunswick, Canada | INA | - [[1](#_ENREF_1)] | partial* | [[6](#_ENREF_6)] |
| M10935-11 | Quebec, Canada | Brook trout | - [[1](#_ENREF_1)] | - | [[6](#_ENREF_6)] |
| M15448-11 | Quebec, Canada | Brook trout | - [[1](#_ENREF_1)] | - | [[6](#_ENREF_6)] |
| M16474-11 | Quebec, Canada | Brook trout | - [[1](#_ENREF_1)] | - | [[6](#_ENREF_6)] |
| M19878-11 | Quebec, Canada | Brook trout | - [[1](#_ENREF_1)] | - | [[6](#_ENREF_6)] |
| M11500-11 | Quebec, Canada | Brook trout | - [[1](#_ENREF_1)] | - | [[6](#_ENREF_6)] |
| M16486-11 | Quebec, Canada | Brook trout | - [[1](#_ENREF_1)] | - | [[6](#_ENREF_6)] |
| M13460-11 | Quebec, Canada | Brook trout | - [[1](#_ENREF_1)] | - | [[6](#_ENREF_6)] |
| M13729-11 | Quebec, Canada | Brook trout | - [[1](#_ENREF_1)] | - | [[6](#_ENREF_6)] |
| M14481-11 | Quebec, Canada | Brook trout | + [[1](#_ENREF_1)] | + | [[6](#_ENREF_6)] |
| M15879-11 | Quebec, Canada | Brook trout | + [[1](#_ENREF_1)] | + | [[6](#_ENREF_6)] |
| M17739-11 | Quebec, Canada | Brook trout | + [[1](#_ENREF_1)] | + | [[6](#_ENREF_6)] |
| M13732-11 | Quebec, Canada | Brook trout | + [[1](#_ENREF_1)] | + | [[6](#_ENREF_6)] |
| M17053-11 | Quebec, Canada | Brook trout | + [[1](#_ENREF_1)] | + | [[6](#_ENREF_6)] |
| M15878-11 | Quebec, Canada | Rainbow trout | - [[1](#_ENREF_1)] | - | [[6](#_ENREF_6)] |
| M13182-11 | Quebec, Canada | Atlantic salmon | - [[1](#_ENREF_1)] | - | [[6](#_ENREF_6)] |
| M17735-11 | Quebec, Canada | Brook trout | - [[1](#_ENREF_1)] | - | [[6](#_ENREF_6)] |
| M15576-11 | Quebec, Canada | Brown trout | - [[1](#_ENREF_1)] | - | [[6](#_ENREF_6)] |
| M15469-11 | Quebec, Canada | Brook trout | + [[1](#_ENREF_1)] | + | [[6](#_ENREF_6)] |
| M22710-11 | Quebec, Canada | Brook trout | - [[1](#_ENREF_1)] | - | [[6](#_ENREF_6)] |
| M13764-11 | Quebec, Canada | Brook trout | - [[1](#_ENREF_1)] | - | [[6](#_ENREF_6)] |
| M18076-11 | Quebec, Canada | Lumpfish | - [[1](#_ENREF_1)] | - | [[6](#_ENREF_6)] |
| M23911-11 | Quebec, Canada | Brook trout | + [[1](#_ENREF_1)] | + | [[6](#_ENREF_6)] |
| JF2267 | Switzerland | Arctic char | - [[1](#_ENREF_1)] | - | [[7](#_ENREF_7)] |
| RS 1458 | Ontario, Canada | Rainbow trout | - [[1](#_ENREF_1)] | - | [[8](#_ENREF_8)] |
| RS 1706 | Ontario, Canada | Chinook salmon | - [[1](#_ENREF_1)] | - | [[8](#_ENREF_8)] |
| RS 1835 | Ontario, Canada | Coho salmon | - [[1](#_ENREF_1)] | - | [[8](#_ENREF_8)] |
| RS 534 (A450) | France | INA | - [[1](#_ENREF_1)] | - | [[9](#_ENREF_9)] |
| RS 1752 | Ontario, Canada | Pumpkinseed | - [[1](#_ENREF_1)] | partial† | [[8](#_ENREF_8)] |
| RS 1705 | Ontario, Canada | Brook trout | + [[1](#_ENREF_1)] | + | [[8](#_ENREF_8)] |
| RS 1744 | Ontario, Canada | Coho salmon | - [[1](#_ENREF_1)] | - | [[8](#_ENREF_8)] |
| RS 887 | Russia | Coho salmon | - [[1](#_ENREF_1)] | - | [[8](#_ENREF_8)] |
| M12357-12 | Quebec, Canada | Brook trout | + [[1](#_ENREF_1)] | + | [[8](#_ENREF_8)] |
| M21375-12 | Quebec, Canada | Brook trout | - [[1](#_ENREF_1)] | - | [[8](#_ENREF_8)] |
| M16237-12 | Quebec, Canada | Brook trout | - [[1](#_ENREF_1)] | - | [[8](#_ENREF_8)] |
| M12976-12 | Quebec, Canada | Brook trout | - [[1](#_ENREF_1)] | - | [[8](#_ENREF_8)] |
| M22895-12 | Quebec, Canada | Brook trout | - [[1](#_ENREF_1)] | - | [[8](#_ENREF_8)] |
| M10745-12 | Quebec, Canada | Brown trout | + [[1](#_ENREF_1)] | + | [[8](#_ENREF_8)] |
| M9754-12 | Quebec, Canada | Brook trout | - [[1](#_ENREF_1)] | - | [[8](#_ENREF_8)] |
| M17930-12 | Quebec, Canada | Brook trout | + [[1](#_ENREF_1)] | + | [[8](#_ENREF_8)] |
| M12418-12 | Quebec, Canada | Brook trout | - [[1](#_ENREF_1)] | - | [[8](#_ENREF_8)] |
| M21368-12 | Quebec, Canada | Brook trout | - [[1](#_ENREF_1)] | - | [[8](#_ENREF_8)] |
| M13050-12 | Quebec, Canada | Brook trout | - [[1](#_ENREF_1)] | - | [[8](#_ENREF_8)] |
| M24783-12 | Quebec, Canada | Brook trout | - [[1](#_ENREF_1)] | - | [[8](#_ENREF_8)] |
| M13566-12 | Quebec, Canada | Brook trout | + [[1](#_ENREF_1)] | + | [[8](#_ENREF_8)] |
| M14404-12 | Quebec, Canada | Atlantic salmon | - [[1](#_ENREF_1)] | - | [[8](#_ENREF_8)] |
| M16671-12 | Quebec, Canada | Brook trout | - [[1](#_ENREF_1)] | - | [[8](#_ENREF_8)] |
| M16042-12 | Quebec, Canada | Brook trout | + [[1](#_ENREF_1)] | + | [[8](#_ENREF_8)] |
| JF2506 | Norway | Atlantic salmon | - [[1](#_ENREF_1)] | - | [[10](#_ENREF_10), [11](#_ENREF_11)] |
| JF2507 | Scotland, United Kingdom | Atlantic salmon | - [[1](#_ENREF_1)] | - | [[10](#_ENREF_10), [11](#_ENREF_11)] |
| JF2510 | Norway | Atlantic salmon | - [[1](#_ENREF_1)] | - | [[10](#_ENREF_10), [11](#_ENREF_11)] |
| JF3224 | Switzerland | Brown trout | - [[1](#_ENREF_1)] | - | [[10](#_ENREF_10), [12](#_ENREF_12)] |
| JF3327 | Switzerland | Arctic char | - [[1](#_ENREF_1)] | - | [[10](#_ENREF_10)] |
| JF3496 | Switzerland | Brown trout | - [[1](#_ENREF_1)] | - | [[10](#_ENREF_10)] |
| JF3517 | Norway | Turbot | - [[1](#_ENREF_1)] | - | [[10](#_ENREF_10)] |
| JF3518 | Norway | Turbot | - [[1](#_ENREF_1)] | - | [[10](#_ENREF_10)] |
| JF3844 | Switzerland | Arctic char | - [[1](#_ENREF_1)] | - | [[10](#_ENREF_10)] |
| JF2869 | INA | Arctic char | - [[1](#_ENREF_1)] | - | [[13](#_ENREF_13)] |
| JF3223 | Switzerland | White Fish | - [[1](#_ENREF_1)] | - | [[10](#_ENREF_10)] |
| JF3507 (NCIMB 1102) | Wales, United Kingdon | Atlantic salmon | - [[1](#_ENREF_1)] | - | [[10](#_ENREF_10), [11](#_ENREF_11), [14](#_ENREF_14)] |
| JF3519 | Switzerland | Arctic char | - [[1](#_ENREF_1)] | - | [[10](#_ENREF_10)] |
| JF3791 | Switzerland | Arctic char | - [[1](#_ENREF_1)] | - | [[10](#_ENREF_10)] |
| JF4111 | Switzerland | Arctic char | - [[1](#_ENREF_1)] | - | [[13](#_ENREF_13)] |
| JF4112 | Switzerland | Arctic char | - [[1](#_ENREF_1)] | - | [[13](#_ENREF_13)] |
| JF4113 | Switzerland | Arctic char | - [[1](#_ENREF_1)] | - | [[13](#_ENREF_13)] |
| JF4114 | Switzerland | Arctic char | - [[1](#_ENREF_1)] | - | [[13](#_ENREF_13)] |
| SHY13-162 | Quebec, Canada | Brook trout | - | - | [[8](#_ENREF_8)] |
| SHY13-574 | Quebec, Canada | Brook trout | - | - | [[8](#_ENREF_8)] |
| SHY13-1470 | Quebec, Canada | Brook trout | - | - | [[8](#_ENREF_8)] |
| SHY13-2188 | Quebec, Canada | Brook trout | + | + | [[8](#_ENREF_8)] |
| SHY13-2222 | Quebec, Canada | Brook trout | - | - | [[8](#_ENREF_8)] |
| SHY13-2257 | Quebec, Canada | Brook trout | - | - | [[8](#_ENREF_8)] |
| SHY13-2263 | Quebec, Canada | Brook trout | - | - | [[8](#_ENREF_8)] |
| SHY13-2317 | Quebec, Canada | Brook trout | - | - | [[8](#_ENREF_8)] |
| SHY13-2425 | Quebec, Canada | Brook trout | - | - | [[8](#_ENREF_8)] |
| SHY13-2458 | Quebec, Canada | Brook trout | + | + | [[8](#_ENREF_8)] |
| SHY13-2534 | Quebec, Canada | Atlantic salmon | - | - | [[8](#_ENREF_8)] |
| SHY13-2627 | Quebec, Canada | Brook trout | - | - | [[8](#_ENREF_8)] |
| SHY13-2630 | Quebec, Canada | Brook trout | + | + | [[8](#_ENREF_8)] |
| SHY13-2825 | Quebec, Canada | Brook trout | - | - | [[8](#_ENREF_8)] |
| SHY13-2873 | Quebec, Canada | Brook trout | - | - | [[8](#_ENREF_8)] |
| SHY13-2909 | Quebec, Canada | Brook trout | - | - | [[8](#_ENREF_8)] |
| SHY13-3101 | Quebec, Canada | Brook trout | + | + | [[8](#_ENREF_8)] |
| SHY13-3127 | Quebec, Canada | Brook trout | - | - | [[8](#_ENREF_8)] |
| SHY13-3795 | Quebec, Canada | Brook trout | + | + | [[8](#_ENREF_8)] |
| SHY13-3798 | Quebec, Canada | Brook trout | - | - | [[8](#_ENREF_8)] |
| SHY13-3799 | Quebec, Canada | Brook trout | - | - | [[8](#_ENREF_8)] |
| SHY14-2246 Rein 1 | Quebec, Canada | Brook trout | - | - | [[8](#_ENREF_8)] |
| SHY14-4161 Furoncle 3 | Quebec, Canada | Brook trout | - | - | [[8](#_ENREF_8)] |
| SHY14-3290 Ec. 2 | Quebec, Canada | Brook trout | + | + | [[8](#_ENREF_8)] |
| SHY14-3402 Rein 1 | Quebec, Canada | Brook trout | - | - | [[8](#_ENREF_8)] |
| SHY14-2420 Rein 1 | Quebec, Canada | Brook trout | - | - | [[8](#_ENREF_8)] |
| SHY14-1503 Rein 1 | Quebec, Canada | Brook trout | - | - | [[8](#_ENREF_8)] |
| SHY14-710 Rein 2 | Quebec, Canada | Brook trout | - | - | [[8](#_ENREF_8)] |
| SHY14-2996 Rein 1 | Quebec, Canada | Arctic char | - | - | [[8](#_ENREF_8)] |
| SHY14-2939 Rein | Quebec, Canada | Brook trout | + | + | [[8](#_ENREF_8)] |
| SHY14-3674 Rein 2 | Quebec, Canada | Brook trout | - | - | [[8](#_ENREF_8)] |
| SHY14-3502 Rein 2 | Quebec, Canada | Brook trout | - | - | [[8](#_ENREF_8)] |
| SHY14-2928 Rein 2 | Quebec, Canada | Brook trout | - | - | [[8](#_ENREF_8)] |
| SHY14-2485 Rein 2 | Quebec, Canada | Brook trout | - | - | [[8](#_ENREF_8)] |
| Shy-15-1459 rein 4 | Quebec, Canada | Rainbow trout | - | - | This study |
| Shy-15-1543 rein 1 | Quebec, Canada | Brook trout | - | - | This study |
| Shy-15-1846 rein 1 | Quebec, Canada | Brook trout | + | + | This study |
| Shy-15-1847 rein 1 | Quebec, Canada | Arctic char | + | + | This study |
| Shy-15-2589 ec. 2 | Quebec, Canada | Arctic char | - | - | This study |
| Shy-15-2743 rein 1 | Quebec, Canada | Rainbow trout | - | - | This study |
| Shy-15-1998 rein 1 | Quebec, Canada | Brook trout | - | - | This study |
| Shy-15-2405 rein 1 | Quebec, Canada | Brook trout | - | - | This study |
| Shy-15-2407 rein 2 | Quebec, Canada | Brook trout | - | - | This study |
| Shy-15-2461 rein 1 | Quebec, Canada | Salmon | - | - | This study |
| Shy-15-2816 rein 2 | Quebec, Canada | Brook trout | - | - | This study |
| Shy-15-2951 rein 1 | Quebec, Canada | Brook trout | - | - | This study |
| Shy-16-4166 rein 1 | Quebec, Canada | INA | + | + | This study |
| Shy-16-4688 ec. 1 | Quebec, Canada | INA | - | - | This study |
| Shy-15-5108 rein 3 | Quebec, Canada | Brook trout | - | - | This study |
| Shy-15-3738 rein 1 | Quebec, Canada | Brook trout | - | - | This study |
| Shy-15-4029 ec. 1 | Quebec, Canada | Brook trout | + | + | This study |
| Shy-15-3379 rein 1 | Quebec, Canada | Brook trout | - | - | This study |
| Shy-15-3412 furoncle 1 | Quebec, Canada | Brook trout | + | + | This study |
| Shy-15-3138 rein 3 | Quebec, Canada | Brook trout | + | + | This study |
| Shy-15-3292 rein 3 | Quebec, Canada | Brook trout | - | - | This study |
| Shy-15-2950 rein 3 | Quebec, Canada | Brook trout | - | - | This study |

*Positive for orf25 only (Table S1)

†Positive for orf25 and orf49

‡ Information given as additional data by the strains’ donors

References

1. Emond-Rheault JG, Vincent AT, Trudel MV, Brochu F, Boyle B, Tanaka KH et al: Variants of a genomic island in *Aeromonas salmonicida* subsp. *salmonicida* link isolates with their geographical origins. Vet Microbiol. 2015;175(1):68-76.

2. Daher RK, Filion G, Tan SG, Dallaire-Dufresne S, Paquet VE, Charette SJ: Alteration of virulence factors and rearrangement of pAsa5 plasmid caused by the growth of *Aeromonas salmonicida* in stressful conditions. Vet Microbiol. 2011;152(3-4):353-360.

3. Dautremepuits C, Fortier M, Croisetiere S, Belhumeur P, Fournier M: Modulation of juvenile brook trout (*Salvelinus fontinalis*) cellular immune system after *Aeromonas salmonicida* challenge. Vet Immunol Immunopathol. 2006;110(1-2):27-36.

4. Popoff M: Etude sur les *Aeromonas salmonicida*. II. Caractérisation des bactériophages actifs sur les *Aeromonas salmonicid*a et lysotypie. Ann Rech Vet. 1971;2(1):33-45.

5. Reith ME, Singh RK, Curtis B, Boyd JM, Bouevitch A, Kimball J et al: The genome of *Aeromonas salmonicida* subsp. *salmonicida* A449: insights into the evolution of a fish pathogen. BMC genomics. 2008;9:427.

6. Trudel MV, Tanaka KH, Filion G, Daher RK, Frenette M, Charette SJ: Insertion sequence *AS5* (IS*AS5*) is involved in the genomic plasticity of *Aeromonas salmonicida*. Mob Genet Elements. 2013;3(3):e25640.

7. Braun M, Stuber K, Schlatter Y, Wahli T, Kuhnert P, Frey J: Characterization of an ADP-ribosyltransferase toxin (AexT) from *Aeromonas salmonicida* subsp *salmonicida*. J Bacteriol. 2002;184(7):1851-1858.

8. Attéré SA, Vincent AT, Trudel MV, Chanut R, Charette SJ: Diversity and Homogeneity among Small Plasmids of *Aeromonas salmonicida* subsp. *salmonicida* Linked with Geographical Origin. Frontiers in microbiology. 2015;6:1274.

9. Kay WW, Buckley JT, Ishiguro EE, Phipps BM, Monette JPL, Trust TJ: Purification and disposition of a surface protein associated with virulence of *Aeromonas salmonicida*. J Bacteriol. 1981;147(3):1077-1084.

10. Burr SE, Frey J: Analysis of type III effector genes in typical and atypical *Aeromonas salmonicida*. J Fish Dis. 2007;30(11):711-714.

11. Olivier G, Moore AR, Fildes J: Toxicity of *Aeromonas salmonicida* cells to Atlantic salmon *Salmo salar* peritoneal macrophages. Dev Comp Immunol. 1992;16(1):49-61.

12. Burr SE, Pugovkin D, Wahli T, Segner H, Frey J: Attenuated virulence of an *Aeromonas salmonicida* subsp. *salmonicida* type III secretion mutant in a rainbow trout model. Microbiology. 2005;151(Pt 6):2111-2118.

13. Studer N, Frey J, Vanden Bergh P: Clustering subspecies of *Aeromonas salmonicida* using IS*630* typing. BMC Microbiol. 2013;13:36.

14. Kupfer M, Kuhnert P, Korczak BM, Peduzzi R, Demarta A: Genetic relationships of *Aeromonas* strains inferred from 16S rRNA, *gyrB* and *rpoB* gene sequences. Int J Syst Evol Microbiol. 2006;56(Pt 12):2743-2751.
